# Emulating Solar Spectral Reflectance of Natural Leaf with Bionic Leaf Prepared from 4A Zeolite-Derived Ultramarine Green Pigment

**DOI:** 10.3390/ma14061406

**Published:** 2021-03-14

**Authors:** Chenglong Lv, Mei Zu, Dongjin Xie, Haifeng Cheng

**Affiliations:** Science and Technology on Advanced Ceramic Fibers and Composites Laboratory, College of Aerospace Science and Engineering, National University of Defense Technology, Changsha 410073, China; lvchenglong@nudt.edu.cn (C.L.); xiedongjin16@nudt.edu.cn (D.X.); chf.cfc@gmail.com (H.C.)

**Keywords:** bionic leaf, green pigment, solar reflectance, 4A zeolite, ultramarine green

## Abstract

The emulation of the reflectance of green leaf in the solar spectral band (300–2500 nm) has garnered increasing attention from researchers. Currently, various materials have been proposed and investigated as potential bionic leaves. However, the problems such as poor weather durability, heavy metal pollution, and complex preparation technology still persist. Herein, a bionic leaf is prepared from an ultramarine green pigment as the functional material, polyvinylidene fluoride (PVDF) as the film-forming material, and LiCl as the humidizer. To prepare the ultramarine green pigment, the sulfur anion is added into the β cage of the 4A zeolite. The mechanisms and properties were discussed based on X-ray diffraction (XRD), scanning electron microscopy (SEM), Raman spectroscopy, and spectroscopic methods. The results show that the as-fabricated bionic leaf based on the 4A zeolite-derived ultramarine green pigment was able to demonstrate a high spectral similarity coefficient of 0.91 with the green leaf. Furthermore, the spectral similarity coefficient was increased to 0.94 after being subjected to a simulated rainforest environment for 48 h, which indicated its high weather durability.

## 1. Introduction

In the field of biomimetics, learning from animals and plants can improve our understandings of nature and create greater convenience in our lives. In recent years, an increasing number of researchers have placed significant attention on the spectral reflectance of green leaf in the solar spectral band to gain insights into itde application during wartime [1,2]. For example, a sniper can utilize the green vegetation as clothing to match the environmental background to avoid detection, etc. Even though the clothes made with plant leaves can confer excellent camouflage ability to the user, however, these clothes are typically bulky and cannot be used during prolonged operations. Thus, researching and developing a biomimetic material that can match the solar spectral reflectance of green vegetation with good durability is very necessary. 

**Figure 1:**
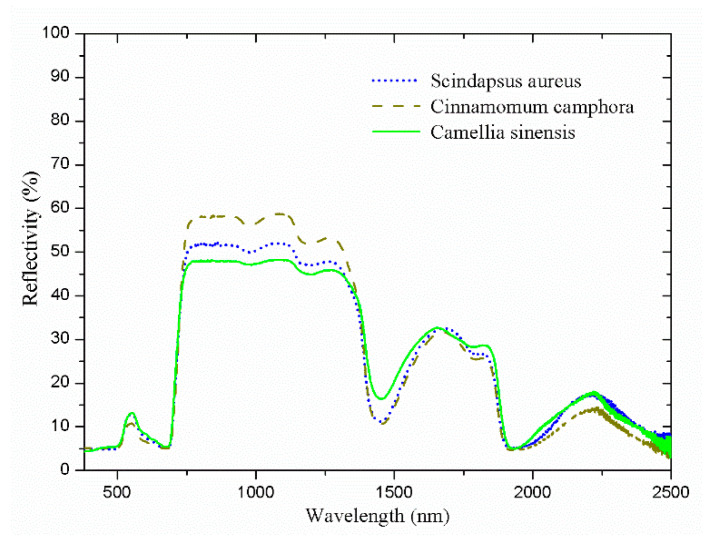
Solar spectral reflectance of *Scindapsus aureus*, *Cinnamomum camphora*, and *Camellia sinensis* leaves.

The solar spectral reflectance characteristics of natural leaves have been widely reported in previous literature [1,3]. The solar spectral reflectance of three different green leaves is shown in Figure 1. Four main characteristics can be observed in the spectral reflectance profile: Firstly, the “green peak” near 550 nm is mainly attributed to the absorption characteristics of chlorophyll in the visible light band [4,5]; secondly, there is a ‘‘red edge’’ at the wavelength of approximately 700 nm. This is because the chlorophyll exhibits a strong absorption peak at 680 nm, and the leaf has a high reflectivity due to its multilayer cell structure at this band [6]. As such, the reflectivity in the spectrum increases suddenly; thirdly, the ‘‘near-infrared plateau’’ region is caused by the scattering process occurring at the multilayer cell structure. The transmittance at this band is uniform, and there is no absorption factor [7]; lastly, there are obvious absorption peaks around 1450 nm and 1940 nm that can be attributed to the presence of water since plant typically contains high water content of approximately 60–80% [8]. Other than the four above-mentioned points during the emulation of the solar spectral reflectance profile of green leaf, life span, and cost should also be considered in the design of a bionic leaf.

The design of the bionic green vegetation leaf can be divided into two types—multilayer structural coating and composite coating. For multilayer structural coating, Liu et al. [1] prepared a multilayer material based on a bionic camouflage material model comprising of an angiosperm leaf. The multilayer material is comprised of a high transmittance film, water-absorbent resin urethane foam, and sodium copper chlorophyllin. Based on their findings, the reflectance spectra of the multilayer material and the Chinese Parasol showed over 96.9% in comparison. Yang et al. [9,10] also developed a four-layer structure that is composed of a transparent polyvinyl chloride (PVC) film, chlorophyll-coated polyvinyl alcohol (PVA) film, a PVC sealed bag containing water, and paper. It was shown that the spectral reflectance profile of the as-prepared multilayer material was similar to that of the camphor leaf. On the other hand, for composite coating, Gao et al. [11] prepared a biomimetic material comprising of a hygroscopic material and Cr_2_O_3_ to counter the hyperspectral detection through the simulation of the solar spectral reflection of a natural leaf. Furthermore, Xu et al. [3] reported two types of biomimetic materials, whereby one utilized Cr_2_O_3_ particles, while the other contained chlorophyll and TiO_2_ particles. The results showed that both biomimetic materials were able to emulate the solar spectral reflectance characteristics exhibited by the leaf. In summary, based on the collective studies, the use of existing materials to emulate the solar spectral reflectance characteristics of green leaves can be achieved effectively. However, there exist common issues with the previously reported bionic leaves, such as complex preparation process, environmental pollution, poor weather durability, etc., which requires urgent resolutions.

Zeolite is a type of microporous material consisting of TO_4_ (T = Al, Si), which can be loaded with functional materials. Previously, 3A zeolites have been incorporated into composite films and showed excellent broadband anti-reflection over the visible–near-infrared wavelength range [12,13]. Moreover, the nanoparticle-loaded 4A-zeolite was used to prepare the PVA/gelatin films, which may be used as an environmentally friendly alternative to plastic packaging materials in the food industries [14]. In addition, we also reported that the 4A zeolite is a promising energy-saving material for daytime passive radiative cooling [15]. However, zeolite has rarely been reported as a bionic material.

In this study, we have synthesized an ultramarine green pigment that is based on 4A zeolite by adding sulfur anion into its β cage (the β cages are built by two secondary building units, every cage is formed by connecting eight six-membered rings and six four-membered rings, assembled as a truncated octahedron) and adjusting the percentage of Na_2_CO_3_ content. The effects of various Na_2_CO_3_ content on the phase structure, morphology, and spectral reflectance are studied. Then, the as-prepared ultramarine green pigment is used to fabricate the bionic leaf and its spectral similarity coefficient to the natural leaf is evaluated. Furthermore, the changes in the solar spectral reflectance profile of the bionic leaf are studied under a simulated rainforest environment. Based on the presented findings, it is expected that the bionic leaf prepared from 4A zeolite-derived ultramarine green pigment can be a promising material that can effectively mimic the natural leaf. 

## 2. Materials and Methods

### 2.1. Preparation of Ultramarine Green Pigment

A total of 3 g sulfur (≥99.5%, Aladdin, Shanghai, China), 2.5 g 4A zeolite (≥95%, average size = 2.13 μm, Tianjin Nanhua catalyst Co., Ltd., Tianjin, China), and 0.3 g SiO_2_ powder (99%, average size = 0.93 μm, Aladdin, Shanghai, China) is used as an additive to increase the content of silicon, 0.5 g rosin (≥90%, Cangzhou Lite Equipment Co., Ltd., Cangzhou, China) is added as a reducing agent, and 0 g, 0.2 g, 0.4 g, or 0.6 g Na_2_CO_3_ (99.5%, average size = 4.66 μm, Aladdin, Shanghai, China) were mixed uniformly (the final products prepared with different Na_2_CO_3_ contents are denoted as product M1, M2, M3, and M4, respectively). Then, the mixed powder was placed in a crucible with a lid, and it was later placed in a furnace. Afterward, the mixed powder was heated to 450 °C (heating rate = 5 °C/min), and it was dwelled for 0.5 h. Subsequently, the mixed powder was then heated with the same heating rate to 800 °C, and it was dwelled for another 2 h. After the heating process, the mixed powder was naturally cooled to room temperature in the furnace. The powder was washed five times with boiling sodium sulfite solution (mass concentration = 1.96%), and then it was washed multiple times with deionized water (pH = 7.1, electrical conductivity = 1.5 µS/cm) until the pH of the filtrate reached 9 or less. Finally, the powder was dried in an oven at 100 °C for 5 h to obtain the ultramarine green pigment.

### 2.2. Preparation of the Bionic Leaf

A total of 0.3 g ultramarine green pigment and 0.1 g LiCl was dispersed into 30 mL polyvinylidene fluoride (PVDF) solution (100 g/L, nitrogen methyl pyrrolidone as the solvent) and disposed of by magnetic stirring for 5 h. Afterward, the mixed solution was coated onto a piece of glass. After drying the coated glass at 70 °C for 5 h, the bionic leaf was finally obtained.

### 2.3. Material Characterization

The phases in the pigment were characterized by an X-ray diffractometer (SmartLab, Rigaku, Japan) with Cu Kα radiation of 1.5418-Å wavelength between 5° and 75°. The morphology of the powder was analyzed using a scanning electron microscope (Sigma 300, Zeiss, Jena, Germany). The diffuse reflectivity (0.3–2.5 µm) was acquired by an ultraviolet–visible–near-infrared spectrophotometer with barium sulfate integrating sphere (UV-4100, Hitachi, Tokyo, Japan). The color of the pigment was evaluated by measuring the L*, a*, and b* parameters using a fiber optic spectrometer (OFS-2500, Ocean optics, Dunedin, FL, USA). The Raman spectra were obtained using a Raman microscope (Invia, Renishaw, Gloucester, United Kingdom). The excitation source is a 532 nm single-mode diode Thorus Laser (Laser Quantum, Cheshire, UK). The tropical rainforest environment was simulated using a Constant Climate Chamber (CTHI-150B, STIK, Shanghai, China). The morphology of the surface of the film was investigated under a super-high magnification lens zoom 3D microscope (VHX-2000C, Keyence, Osaka, Japan).

### 2.4. Calculation

The spectral similarity coefficient γ_xy_ was calculated using the following formula [16,17]:

(1) $$\gamma_{\mathrm{xy}}=\frac{\sum_{i=1}^{m}\left(x_{i}-{\bar{x}}_{I}\right)\left(y_{i}-{\bar{y}}_{I}\right)}{\sum_{i=1}^{m}{\left(x_{i}-{\bar{x}}_{I}\right)}^{2}\sum_{i=1}^{m}{\left(y_{i}-{\bar{y}}_{I}\right)}^{2}}$$

where $x_{i}$ is the spectral values of the measured sample, and $y_{i}$ is the spectral values of the reference substance at a wavelength of I. ${\bar{x}}_{I}=\frac{1}{m}\sum_{i=1}^{m}x_{i}\;\mathrm{and}\;{\bar{y}}_{I}=\frac{1}{m}\sum_{i=1}^{m}y_{i}$ are the mean spectral values of the measured sample and the reference substance, respectively. According to the formula, the spectral reflectance of the biomimetic material and plant leaf in the region of 300–2500 nm can have a similarity coefficient of up to 1, which indicates that the biomimetic material exhibits similar reflectance characteristics as the green leaf.

## 3. Results

**Figure 2:**
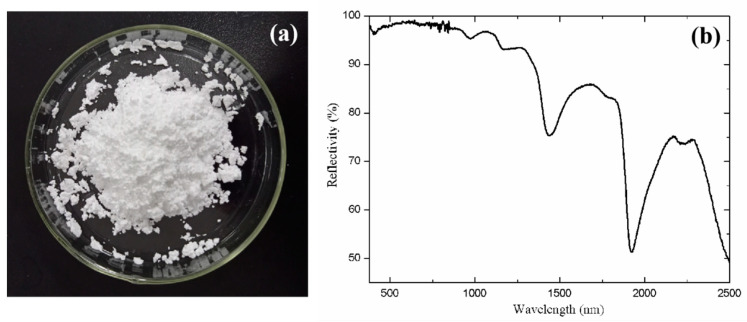
(**a**) Optical photograph and (**b**) solar spectral reflectance profile of the 4A zeolite.

Figure 2 shows the digital photograph and the solar spectral reflectance profile of the 4A zeolite within the visible and near-infrared range. As shown in Figure 2a, the color of 4A zeolite is white, and it does not show obvious absorption within the visible light band (Figure 2b). In the near-infrared band (780–2500 nm), the spectral reflectance profile of the material is consistent with that of the green leaf. This is because 4A zeolite possesses a strong water adsorption ability. According to the spectral similarity coefficient result, the correlation coefficient is 0.60 in the visible light band, while it is 0.95 when only considering the near-infrared band. This result indicates that 4A zeolite possesses great potential in emulating the water absorption peaks that are typically observed for the green vegetation. It is worth noting that the distinction between the 4A zeolite and the green vegetation lies in the spectral reflectance profile at the visible light band. Thus, in the following study, we will add chromogenic groups into the 4A zeolite to improve its visible light spectrum, and we will also use the inorganic anionic sulfur instead of metal ion to mitigate the environmental issues. 

The temperature of the heat treatment process and the ratio of sodium to sulfur (Na_2_/S) in the raw materials are the main factors that can affect the composition and reflectance spectrum of the final product [18]. The temperature of the heat treatment process is set at 800 °C, while the mass of sodium carbonate varies from 0 g, 0.2 g, 0.4 g, and 0.6 g. With this systematic investigation, the optimal sample is later selected for the subsequent investigation of its emulation on the solar reflectance spectrum of the natural leaf. 

**Figure 3:**
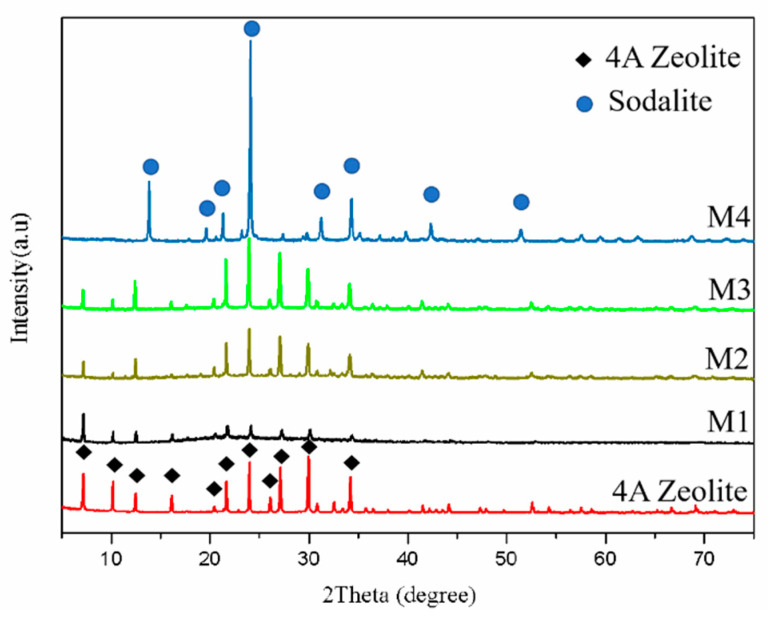
X-ray diffraction (XRD) spectra of 4A zeolite and various materials with different Na_2_CO_3_ contents.

According to Figure 3, the XRD spectra of the product M1–M3 are consistent with that of 4A zeolite. This result suggests that the Linde-A phase can still be retained after adding Na_2_CO_3_ content of less than 0.4 g with subsequent heat treatment at 800 °C. However, the peaks of product M1 are weaker than those of product M2 and product M3. When the Na_2_CO_3_ content increases to 0.6 g, the material changes to lazurite, whereby the phase transformed from the LTA phase (4A zeolite) to the Sodalite phase (lazurite) [19,20]. 

**Figure 4:**
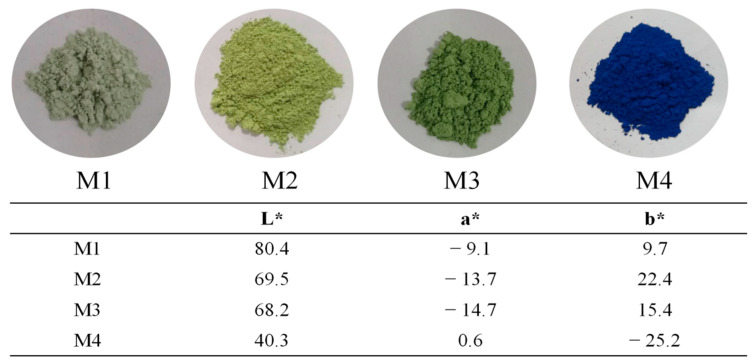
Photographs and colorimetric parameters (L*, a*, b*) of the products.

The phase transition of the products corresponds to a change in color. Figure 4 shows the digital photographs of the four products with varying amounts of Na_2_CO_3_ contents. As shown clearly in the digital photograph, the color of product M1 is light green. This color exhibited by product M1 may be caused by the lack of Na_2_CO_3_. On the other hand, product M2 is yellow, and product M3 is green with their respective b* values of 22.4 and 15.4, which means that product M2 contains a much greater yellow color component than product M3. For product M4, it is clearly observed that it appears blue with L*, a*, b* value of 40.3, 0.6, and −25.2, respectively. This change in color of the ultramarine can be attributed to the relative contents of S_2_^−^ and S_3_^−^ anion in the cage [21]. It is worth noting that S_2_^−^ corresponds to yellow and S_3_^-^ corresponds to blue; therefore, the presence of both S_2_^−^ and S_3_^−^ can lead to the mixture of yellow and blue to produce green [22]. In this study, with the increase of the Na_2_CO_3_ content, the concomitant increase in the alkalinity can result in an increase in S_3_^−^ content, therefore causing the color of the product to change from yellow to green and then to blue [23]. 

**Figure 5:**
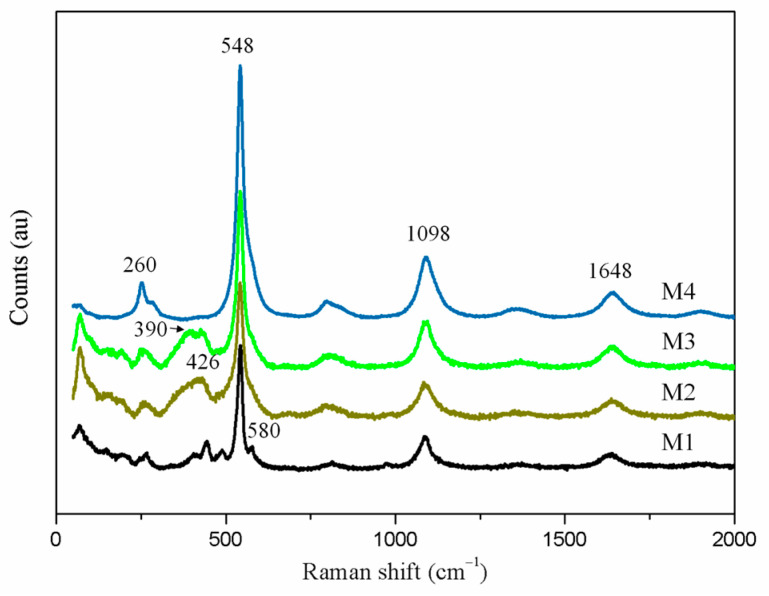
Raman spectra of product M1, M2, M3, and M4.

Raman spectroscopy is an effective method used to distinguish the different sulfur ions, and it has been widely applied in the study of ultramarine pigments [24]. Figure 5 shows the Raman spectra of the various products. The peak at 548 cm^−1^ can be observed for all products, which can be caused by the symmetric stretching vibration of S_3_^−^ radical [25]. The peaks located at 1098 cm^−1^ and 1648 cm^−1^ can be ascribed to the first and second overtones of the S_3_^−^ stretching mode, respectively. Furthermore, the peak located at 260 cm^−^^1^ is caused by the bending vibration of S_3_^−^ [26]. The peak for S_2_^−^ is usually located at 580 cm^−1^, which can be observed in product M1. However, this peak cannot be clearly observed for other products since it may be masked by the strong S_3_^−^ peak located at 548 cm^−1^. In addition, obvious differences at the wavenumber of 390 cm^−1^ and 426 cm^−1^ can be observed across the products, which are mainly caused by the phase change.

**Figure 6:**
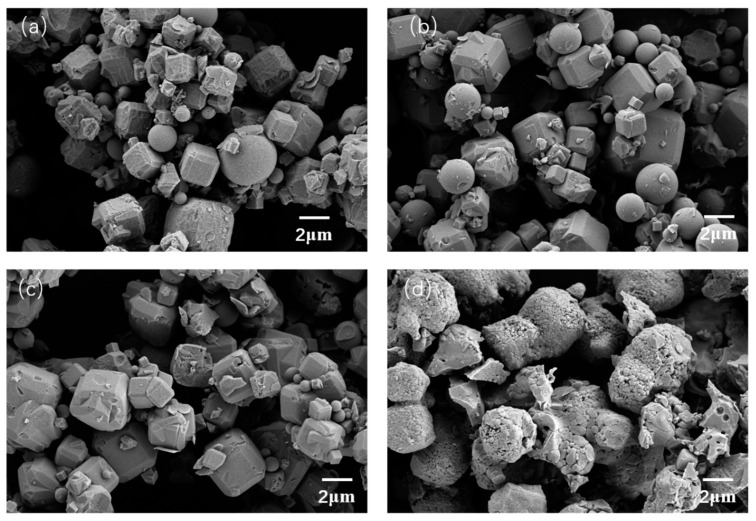
Scanning electron microscopy (SEM) images of the products prepared with different Na_2_CO_3_ content (**a**) 0 g; (**b**) 0.2 g; (**c**) 0.4 g; and (**d**) 0.6 g.

Figure 6 shows the SEM images of the products, reveal particles with similar morphology (consists of cubic and round particles) for product M1, M2, and M3, whereby the cubic particle is the 4A zeolite. However, the morphology of the particle exhibits a significant change when 0.6 g sodium carbonate is added. During this situation, the particles are irregular particles or agglomerate formed by numerous nanoparticles. Although the phase of the material changes, the agglomerated particles of M4 still possess a similar size to products M1, M2, and M3.

**Figure 7:**
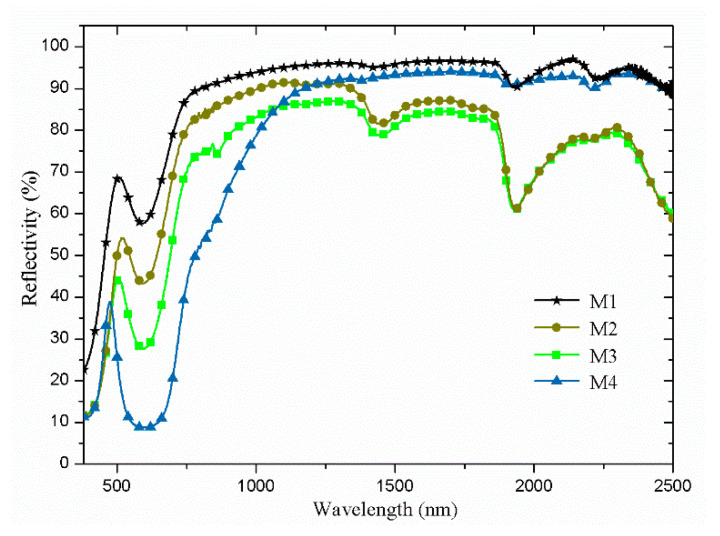
Spectral reflectance profiles of various products.

Figure 7 shows the spectral reflectance profiles of the four synthesized products. The reflection peaks appear at 510 nm, 517 nm, 508 nm, and 468 nm, which correspond to the L* value of 80.4, 69.5, 68.2, and 40.3 for product M1, M2, M3, and M4, respectively. At the “red edge”, the reflectivity is quite different, and this may be caused by their different phases and morphologies. At the wavelength over 1000 nm, products M2 and M3 show obvious water absorption peaks at the wavelength of 1450 nm and 1940 nm. This is largely due to the structure of the LTA phase (Lind type A zeolite), whereby LTA possesses a pore size of 0.4 nm that is similar to the size of the water molecule [15]. However, the water absorption peaks are not observed for products M1 and M4. For product M1, this may be caused by the pore structure of 4A zeolite being blocked after subjecting to heat treatment at 800 °C, which results in the weakening of its water adsorption ability. However, for product M4, the lack of water absorption peak is mainly due to the phase change. The calculated spectral similarity coefficients of product M1, M2, M3, and M4 to green vegetation leaf are 0.39, 0.69, 0.89, and 0.16, respectively. Thus, based on this result, product M3 exhibits the greatest potential in the emulation of the spectral reflectance profile of green vegetation leaf. 

**Figure 8:**
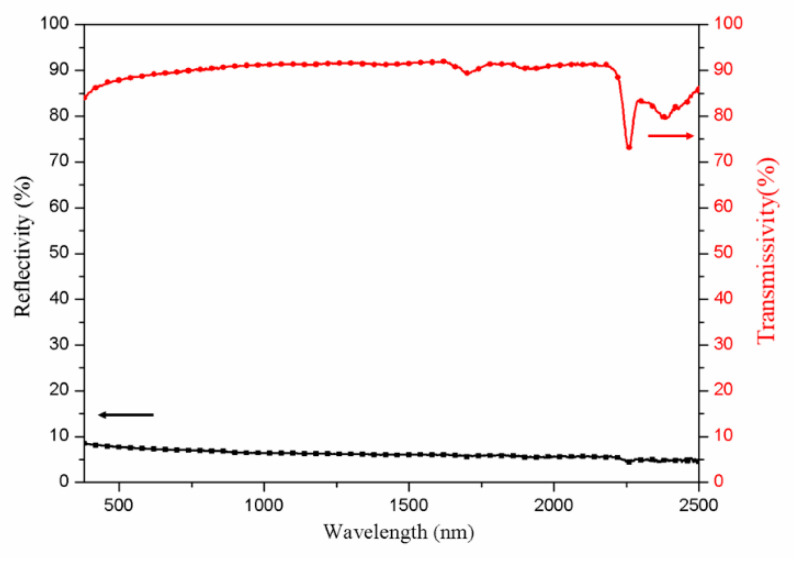
Spectral reflectance and transmittance profiles of polyvinylidene fluoride (PVDF) film.

In the preparation of bionic leaf, product M3 is chosen as the pigment, and PVDF is used as a film-forming material. Before the preparation of the bionic leaf, the spectral reflectance and transmittance profiles of PVDF film are first investigated. As shown in Figure 8, the PVDF film has a low reflectivity of ~6% and a high transmissivity of ~90%. At the wavelength of ~2250 nm, the transmissivity curve has a weak absorption peak, and this is mainly caused by the combined absorption of the chemical bonds –CH_2_ [27,28]. This result suggests that the influence of PVDF on the functionality of the filler, when used as the film-forming material, is negligible, and as such its effect could be neglected.

**Figure 9:**
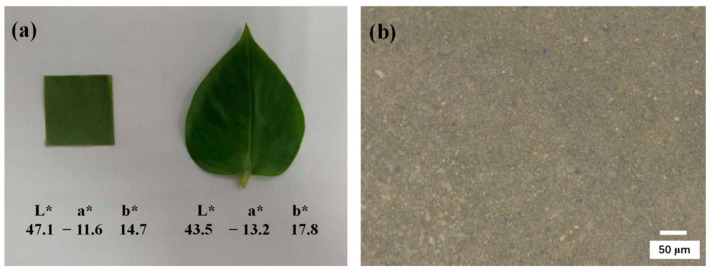
(**a**) Photograph and colorimetric parameters (L*, a*, b*) of bionic film and *Scindapsus aureus* leaf (thickness = 146 μm) and (**b**) micrograph of the bionic film.

The digital photograph and the micrograph of the as-fabricated bionic leaf are shown in Figure 9. According to the photograph, the bionic leaf has a similar L*, a*, b* value as the *Scindapsus aureus* leaf (Figure 9a). Furthermore, based on the micrograph, it can be observed that the surface of the sample is smooth and even (Figure 9b).

**Figure 10:**
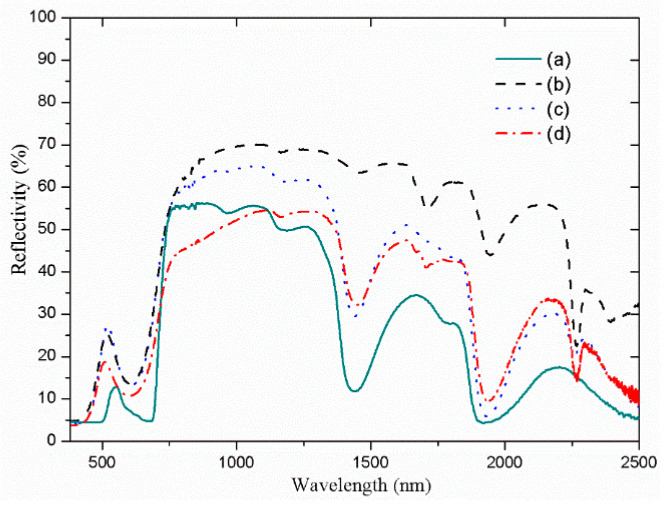
Solar spectral reflectance profiles of various materials: (**a**) *Scindapsus aureus*; (**b**) bionic leaf A synthesized with PVDF and product M3; (**c**) bionic leaf B synthesized with PVDF, LiCl, and product M3; and (**d**) bionic leaf B after a simulated rain forest environmental condition for 48 h.

Figure 10 shows the solar spectral reflectance profiles of various materials, which include *Scindapsus aureus* and the as-prepared films. According to the spectral reflectance profiles, the reflectance spectrum of the bionic leaf A is roughly consistent with that of the *Scindapsus aureus* leaf. However, with closer inspection, the visible light band and the water absorption peaks at 1450 nm and 1940 nm for these two materials are relatively different. These differences may arise due to the weaker adsorption ability at the wavelength of 680 nm and the lower water content in the bionic leaf A than those in the actual leaf. Thus, in order to increase the water content and water adsorption ability in the bionic leaf, a super absorbent salt, i.e., LiCl, is selected as an auxiliary component, and the spectral reflectance profile of this synthesized bionic leaf is shown in Figure 10c. In this case, the peak at 2250 nm that is caused by PVDF is weakened, while the peaks at 1450 nm and 1940 nm are enhanced. The spectral similarity coefficient of the bionic leaf with LiCl to *Scindapsus aureus* increases from an initial 0.73 (bionic leaf A) to 0.91. Thus, based on these results, a composite film material with ultramarine green pigment, LiCl as functional filler, and PVDF as film-forming material can show excellent emulation to the natural *Scindapsus aureus*. Furthermore, to increase the practicality of the as-prepared bionic leaf, its weather durability is also investigated under a simulated rain forest environment (humidity = 90%, temperature = 30 °C). To emulate the rain forest condition, the bionic leaf B is placed in the Constant Climate Chamber for 48 h, and the spectral reflectance profile is subsequently collected. As shown in Figure 10d, the bionic leaf, after subjected to 48 h of simulated rain forest condition, exhibits a spectral similarity coefficient of as high as 0.94. This result clearly demonstrates the excellent durability of the as-prepared bionic leaf in harsh weather conditions. At the same time, it is also noted that the difference in the spectra of the bionic leaf and the green leaf, especially at the “green peak” and the “red edge,” requires further study in the future.

## 4. Conclusions

To emulate the solar spectral reflectance of the green vegetation leaf, a bionic leaf was prepared from a 4A zeolite-derived ultramarine green pigment. It was shown in this study that due to its water-absorbing ability, the 4A zeolite possessed a similar spectral reflectance profile as the green leaf within a wavelength range of 1000 nm to 2500 nm, with a spectral similarity coefficient of 0.95. At the visible light band, the color of 4A zeolite can be altered by adding the sulfur anion into the cage of the LTA phase. It was shown that the product exhibited a similar spectral profile with the green leaf when the mass of reactant—Na_2_CO_3_—is 0.4 g. Finally, a bionic leaf with a spectral similarity coefficient of 0.91 to the natural leaf was obtained in this study that comprised of the ultramarine green pigment, PVDF, and LiCl. Furthermore, the bionic leaf film exhibited a spectral similarity coefficient of 0.94 even after being subjected to a simulated rainforest environment for 48 h. Thus, based on the collective results, the as-prepared bionic leaf from the 4A zeolite-based pigment shows excellent emulation to the solar spectral reflectance profile of the green leaf.

## Data Availability

The processed data required to reproduce these findings cannot be shared at this time as the data also forms part of an ongoing study.

## References

[1] Liu Z.M., Hu B.R., Wu W.J., Zhang Y. (2009). Spectral imaging of green coating camouflage under hyperspectral detection. Guangzi Xuebao/Acta Photonica Sin..

[2] Pauli D., Chapman S.C., Bart R., Topp C.N., Lawrence-Dill C.J., Poland J., Gore M.A. (2016). The quest for understanding phenotypic variation via integrated approaches in the field environment. Plant Physiol..

[3] Xu K., Ye H. (2020). Preparation and optimization of biomimetic materials simulating solar spectrum reflection characteristics of natural leaves. J. Mater. Sci..

[4] Sims D.A., Gamon J.A. (2002). Relationships between leaf pigment content and spectral reflectance across a wide range of species, leaf structures and developmental stages. Remote Sens. Environ..

[5] Maas S.J., Dunlap J.R. (1989). Reflectance, Transmittance, and Absorptance of Light by Normal, Etiolated, and Albino Corn Leaves. Agron. J..

[6] Knipling E.B. (1970). Physical and physiological basis for the reflectance of visible and near-infrared radiation from vegetation. Remote Sens. Environ..

[7] Gates D.M., Keegan H.J., Schleter J.C., Weidner V.R. (1965). Spectral Properties of Plants. Appl. Opt..

[8] Peñuelas J., Inoue Y. (1999). Reflectance indices indicative of changes in water and pigment contents of peanut and wheat leaves. Photosynthetica.

[9] Yang Y.J., Hu B.R., Wu W.J. (2011). Design and preparation of bionic camouflage materials by simulating plant leaves. Guofang Keji Daxue Xuebao/J. Natl. Univ. Def. Technol..

[10] Yang Y., Liu Z., Hu B., Man Y., Wu W. (2010). Bionic composite material simulating the optical spectra of plant leaves. J. Bionic Eng..

[11] Gao Y., Ye H. (2017). Bionic membrane simulating solar spectrum reflection characteristics of natural leaf. Int. J. Heat Mass Transf..

[12] Fiorillo A.S., Pullano S.A., Rudenko S.P., Stetsenko M.O., Maksimenko L.S., Krishchenko I.M., Synyuk V.S. (2018). Antireflection properties of composite zeolite gold nanoparticles film. Electron. Lett..

[13] Stetsenko M., Pullano S.A., Margitych T., Maksimenko L., Hassan A., Kryvyi S., Hu R., Huang C., Ziniuk R., Golovynskyi S. (2019). Antireflection enhancement by composite nanoporous zeolite 3A–carbon thin film. Nanomaterials.

[14] Azizi-Lalabadi M., Alizadeh-Sani M., Divband B., Ehsani A., McClements D.J. (2020). Nanocomposite films consisting of functional nanoparticles (TiO2 and ZnO) embedded in 4A-Zeolite and mixed polymer matrices (gelatin and polyvinyl alcohol). Food Res. Int..

[15] Lv C., Zu M., Xie D., Yan F., Li M., Cheng H. (2020). 4A zeolite based daytime passive radiative cooling material. Infrared Phys. Technol..

[16] Wang J., Li C., Liu L., Zheng S., Xiang T., Yang L. (2017). Design and application of simulation material of green vegetation spectrum in NIR interval. Guangxue Xuebao/Acta Opt. Sin..

[17] Qin R., Xu G., Guo L., Jiang Y., Ding R. (2012). Preparation and characterization of a novel poly(urea-formaldehyde) microcapsules with similar reflectance spectrum to leaves in the UV-Vis-NIR region of 300–2500 nm. Mater. Chem. Phys..

[18] Aranzabe E., Villasante P.M., March R., Arriortua M.I., Vadillo J., Larrañaga A., Aranzabe A. (2016). Preparation and characterization of high NIR reflective pigments based in ultramarine blue. Energy Build..

[19] Finch A.A., Friis H., Maghrabi M. (2016). Defects in sodalite-group minerals determined from X-ray-induced luminescence. Phys. Chem. Miner..

[20] Hassan I., Peterson R.C., Grundy H.D. (1985). The structure of lazurite, ideally Na6Ca2(Al6Si6O24)S2, a member of the sodalite group. Acta Crystallogr..

[21] Kowalak S., Jankowska A. (2009). Inorganic Sulphur Pigments Based on Nanoporous Materials. Ordered Porous Solids.

[22] Cato E., Rossi A., Scherrer N.C., Ferreira E.S.B. (2018). An XPS study into sulphur speciation in blue and green ultramarine. J. Cult. Herit..

[23] Wang H., Zhang S., Hu S., Zhen Z., Gomez M.A., Yao S. (2020). A systematic study of the synthesis conditions of blue and green ultramarine pigments via the reclamation of the industrial zeolite wastes and agricultural rice husks. Environ. Sci. Pollut. Res..

[24] González-Cabrera M., Arjonilla P., Domínguez-Vidal A., Ayora-Cañada M.J. (2020). Natural or synthetic? Simultaneous Raman/luminescence hyperspectral microimaging for the fast distinction of ultramarine pigments. Dye. Pigment..

[25] El Jaroudi O., Picquenard E., Demortier A., Lelieur J.P., Corset J. (2000). Polysulfide anions II: Structure and vibrational spectra of the S42- and S52- anions. Influence of the cations on bond length, valence, and torsion angle. Inorg. Chem..

[26] Colomban P., Tournié A., Caggiani M.C., Paris C. (2012). Pigments and Enamelling/Gilding Technology of Mamluk Mosque Lamps and Bottle. J. Raman Spectrosc..

[27] Workman J., Weyer L. (2012). Practical Guide and Spectral Atlas for Interpretive Near-Infrared Spectroscopy.

[28] Siesler H.W., Ozaki Y., Kawata S., Heise H.M. (2002). Near-Infrared Spectroscopy. Principles, Instruments, Applications.

